# Metachronous Dual Primary Malignancies of Carcinoma of the Tongue and Hodgkin's Lymphoma

**DOI:** 10.7759/cureus.2361

**Published:** 2018-03-23

**Authors:** Pooja Uttam Mate, Kapil Suri

**Affiliations:** 1 Radiotherapy, VMMC & Safdarjung Hospital, New Delhi

**Keywords:** malignancy, lymphoma, chemotherapy, metachronous, hodgkin's disease

## Abstract

Hodgkin's lymphoma in a treated case of carcinoma of the tongue outside the irradiated area is a rare occurrence. Treatment-associated second malignancies have been reported in irradiated patients. Here, we report a case of 34-year-old male who was diagnosed with carcinoma of the tongue in March 2015 and developed Hodgkin's lymphoma in October 2016. Though Hodgkin's lymphoma has been reported in the radiation area after 10 - 15 years, in this case, we encountered Hodgkin's lymphoma after treating a patient with carcinoma of the tongue after one and a half years and outside the irradiated area. To our knowledge, this is the first case of metachronous malignancy with this unusual presentation.

## Introduction

The prevalence of multiple malignancies is increasing with the advent of advanced diagnostic imaging and therapeutic modalities. During follow-up screening of this patient status-post treatment for carcinoma of the tongue, we picked up an early asymptomatic case of Hodgkin's lymphoma. The risk of developing a second malignancy after irradiation is known and has been seen to occur after many years. As per the definition of radiation-induced malignancy, a sufficient latent period, preferably longer than four years, must have elapsed between the initial irradiation and the alleged induced malignancy [1]. However, whether it is radiation-induced or metachronous is debatable. We report one such unusual case of a Stage IA Hodgkin's lymphoma which was outside the irradiated area.

## Case presentation

A 34-year-old male cigarette smoker presented with a non-healing ulcer over the right side of the tongue for three months. On examination, an ulceroproliferative growth was present over the right side of the tongue over the lateral border just behind the tip with no clinically palpable cervical lymph node involvement. The patient underwent a right partial glossectomy with right selective neck dissection (I-IV) in March 2015. His histopathological report showed moderately differentiated squamous cell carcinoma in the right lateral border of the tongue. The tumour measured 2.2 x 2 x 1.8 cm, the depth of invasion was 1.4 of 1.6 cm, margins were free, lymphovascular and perineural invasion were positive, and 45 lymph nodes were isolated, out of which one showed tumor metastasis with extranodal spread (pT2N1). In view of the extracapsular spread, he received concomitant chemoradiotherapy, 60 Gy/30#, along with weekly injections of cisplatin 30 mg/m^2^ (6 megavoltage (MV) photons). The patient completed treatment in May 2015. Thereafter, the patient was asymptomatic and was on regular follow-up.

**Figure 1:**
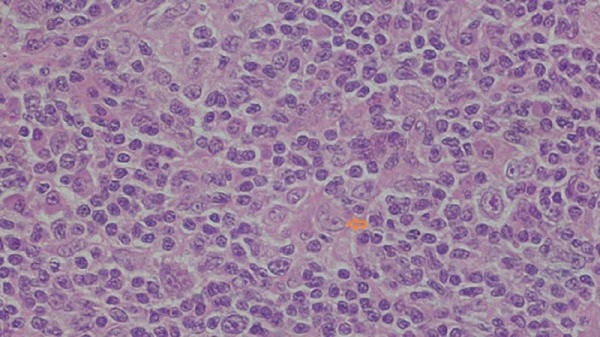
Excisional biopsy consistent with Hodgkin's lymphoma mixed cellularity type The biopsy reveals diffuse effacement of nodal architecture by a polymorphs population comprising of lymphocytes, plasma cells, and eosinophils. There are classical as well as mononuclear variants of Reed Sternberg cells.

Positron emission tomography-computed tomography (PET-CT) done in October 2016 during follow-up showed multiple variable-sized lymph nodes in the right axillary region measuring 23 x 14 mm standardized uptake value (SUV-14) with no residual/recurrent mass at the primary site. Excisional biopsy was done, which was consistent with Hodgkin's lymphoma mixed cellularity type (Figure 1).

**Figure 2:**
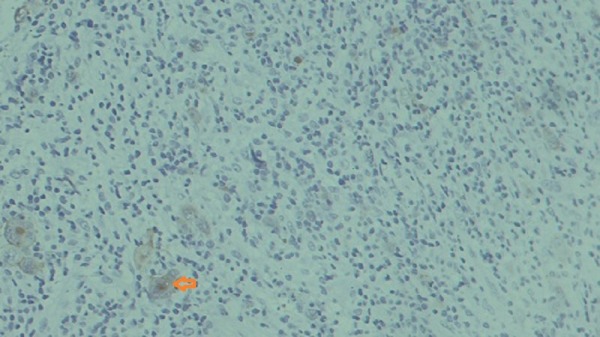
Immunohistochemistry from October 2016 follow-up Immunohistochemistry showing lymphoid cells positive for CD20 and CD3. Reed Sternberg cells were positive for CD15 (focal).

There was diffuse effacement of the nodal architecture by a polymorphs population comprised of lymphocytes, plasma cells, and eosinophils. There were classical as well as the mononuclear variant of Reed Sternberg cells with prominent nucleoli. An extranodal spread was also seen. On immunohistochemistry, lymphoid cells were positive for CD20 and CD3. Reed Sternberg cells were positive for CD15 (focal) ( Figure 2).

The patient was started on chemotherapy with Adriamycin, bleomycin, vincristine, and dacarbazine (ABVD) regimen. He completed three cycles of chemotherapy following which a PET-CT showed Deauville score of 3-4. So again, one more cycle of chemotherapy was administered and involved-field radiotherapy (20 Gy/10#) to the right axilla was given. At present, the patient is asymptomatic and on regular follow-up.

## Discussion

Billroth, in 1889, was the first to report dual malignancy [2]. The tumors may be called as synchronous when a second tumor develops within six months of detection of the first, and metachronous when the second tumor develops more than six months after detection of the first. According to the Warren and Gates criteria (1932) for the diagnosis of a second primary malignancy, both tumors must be confirmed on histopathology, each must be distinct, and the probability of one being a metastasis of the other must be excluded [3].

Hodgkin's lymphoma is usually almost entirely confined to the lymph nodes and the extranodal disease manifests clinically in less than 1% of cases in the head and neck region. In our case, the initial histopathological report of the tongue lesion was a moderately differentiated squamous cell carcinoma, so the possibility of that lesion being an extranodal manifestation of Hodgkin's lymphoma was ruled out. PET integrated with CT is a powerful imaging tool for biochemical, functional, morphological, and anatomical assessment. PET-CT is used for diagnosing the site of an unknown primary, staging, response evaluation, restaging, and image-guided biopsy. It is now an established standard in initial staging, response assessment, and restaging after treatment of patients with Hodgkin's lymphoma and high-grade Hodgkin's lymphoma [4]. It has a 94% sensitivity and 100% specificity for lymph node involvement [5]. In head and neck malignancies, PET-CT detects synchronous primaries in 8.1%, the site of an unknown primary in 73%, and distant metastases in 15.4% [6]. PET-CT has a higher sensitivity and specificity than conventional CT when done after eight weeks of chemoradiotherapy [7]. The radiation-induced second malignant neoplasm can occur within the treatment field or outside the radiation field. The possible mechanism for causing malignancies outside the treatment field is low-dose scatter radiation and bystander effect [8]. In our case, whether Hodgkin's lymphoma was radiation-induced or not is debatable.

## Conclusions

Herein, we report an unusual case of Hodgkin's lymphoma occurring outside the irradiated field which was detected during routine follow-up in an asymptomatic patient with carcinoma of the tongue with the use of a PET-CT scan. Thus, in this case, a PET-CT played an important role during follow-up for diagnosing a second malignancy. A close and regular follow-up plays a crucial role in the early diagnosis of a second malignancy.
